# Ex vivo electrochemical measurement of glutamate release during spinal cord injury

**DOI:** 10.1016/j.mex.2019.08.008

**Published:** 2019-08-23

**Authors:** James K. Nolan, Tran N.H. Nguyen, Mara Fattah, Jessica C. Page, Riyi Shi, Hyowon Lee

**Affiliations:** a Weldon School of Biomedical Engineering, Birck Nanotechnology Center, Center for Implantable Devices, Purdue University, West Lafayette, IN, USA; b Department of Basic Medical Sciences, College of Veterinary Medicine, Purdue University, West Lafayette, IN, USA

**Keywords:** Electrochemical Glutamate Sensing from Resected Spinal Cord Segment, Biosensor, SCI, Excitotoxicity, Direct ink writing, Additive manufacturing, Rapid prototyping, Implantable

## Abstract

Excessive glutamate release following traumatic spinal cord injury (SCI) has been associated with exacerbating the extent of SCI. However, the mechanism behind sustained high levels of extracellular glutamate is unclear. Spinal cord segments mounted in a sucrose double gap recording chamber are an established model for traumatic spinal cord injury. We have developed a method to record, with micro-scale printed glutamate biosensors, glutamate release from *ex vivo* rat spinal cord segments following injury. This protocol would work equally well for similar glutamate biosensors.

## Graphical abstract

**Figure:**
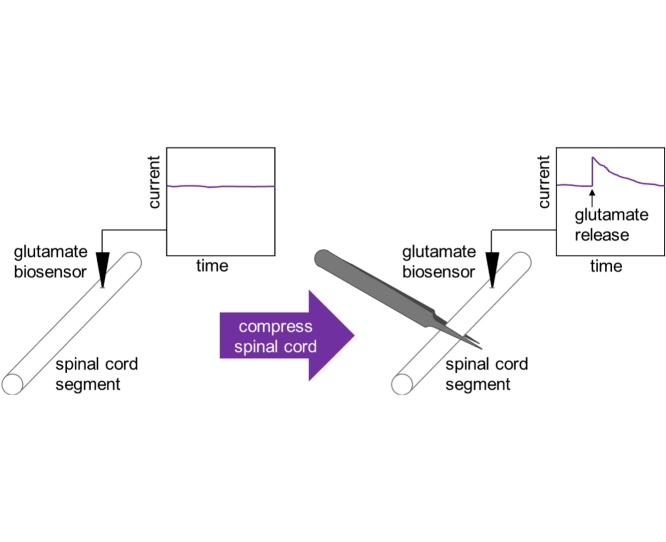

## Specifications Table

**Table:** 

Subject Area:	Neuroscience
More specific subject area:	*Glutamate Excitotoxicity and Its Role in Spinal Cord Injury*
Protocol name:	*Electrochemical Glutamate Sensing from Resected Spinal Cord Segment*
Reagents/tools:	• Bio-Logic SP-200 Potentiostat (Bio-Logic, Knoxville, TN, USA): https://www.bio-logic.net/products/multichannel-conductivity/sp-200-potentiostat-galvanostat/ • EC-Lab® V11.02 software (Bio-Logic, Knoxville, TN, USA): https://www.bio-logic.net/softwares/ec-lab-software/#1461666531286-9f792007-63fe • RE-5B Ag/AgCl (3M NaCl) reference electrode (BASi, Part No. MF-2052, West Lafayette, IN, USA): https://www.bio-logic.net/softwares/ec-lab-software/#1461666531286-9f792007-63fe • Ag/AgCl ink (Engineered Conductive Materials, Inc., Part No. CI-4001, Delaware, OH): http://www.conductives.com/biosensors.php • Pt auxiliary electrode (BASi, Part No. MW-4130, West Lafayette, IN, USA): https://www.basinc.com/products/813 • Hook test lead wires (E-Z Hook, Arcadia, CA, USA): https://catalog.e-z-hook.com/viewitems/test-leads/e-z-micro-hook-test-leads-2 • L-glutamic acid, 99+% (Alfa Aesar, Stock # A15031, Tewksbury, MA, USA): https://www.alfa.com/en/catalog/A15031/ • L-ascorbic acid, 99% (Sigma-Aldrich, Product # A92902, St. Louis, MO, USA): https://www.sigmaaldrich.com/catalog/product/sial/a92902?lang=en&region=US • Phosphate-buffered saline (PBS), 10X (0.1M) pH 7.4 (Thermo Fisher Scientific, Cat. # AM9625, Waltham, MA, USA): https://www.thermofisher.com/order/catalog/product/AM9625 • Cimarec+™ stirring hotplate (Thermo Fisher Scientific, Cat. # SP88857107, Waltham, MA, USA): https://www.thermofisher.com/order/catalog/product/SP88857107?SID=srch-srp-SP88857107 • 1 Mil Kapton® tape (polyimide tape) (Kapton Tape, Part # KPT-1/4, Torrance, CA, USA): https://www.kaptontape.com/1_Mil_Kapton_Tapes.php • Micromanipulator (Newport, 433 series, Irvine, CA, USA) (Fig. 2) • Double sucrose gap recording chamber (Fig. 2) • Minipuls 3 peristaltic pump (Gilson, Middleton, WI, USA): https://www.gilson.com/system-minipuls-3-peristaltic-pumps.html • Fine point forceps (Thermo Fisher Scientific, Cat. # 12-000-122, Waltham, MA, USA): https://www.fishersci.com/shop/products/high-precision-straight-tapered-ultra-fine-point-tweezers-forceps/12000122
Experimental design:	A spinal cord segment was isolated from a rat and maintained in oxygenated Krebs solution. A biosensor was inserted into the spinal cord and recorded glutamate concentration while the spinal cord was compressed to simulate injury.
Trial registration:	n/a
Ethics:	All animals were used according to the Purdue University Animal Care and Use Committee protocol and guidelines.

**Value of the Protocol**

**Table:** 

• Glutamate biosensor measures in real time with better spatial and temporal resolution than microdialysis • Measuring from the spinal cord ex vivo removes processes and variables, such as hemorrhage and ischemia, that may obscure glutamate release • The glutamate biosensors used were made by direct writing of nanocomposite ink, which is an easy and fast fabrication method in comparison to conventional micro-fabrication

## Description of protocol

This protocol records extracellular glutamate with high spatial (100 μm) and temporal (1 s) resolution from a spinal cord segment during injury [1,2]. Glutamate release following traumatic spinal cord injury (SCI) exacerbates the extent of SCI [3], yet the mechanism behind sustained high levels of extracellular glutamate has remained unclear. This protocol can be used to study the relationship between extracellular glutamate and other molecules, such as acrolein, and develop therapeutic interventions [4].

### Materials

• Bio-Logic SP-200 Potentiostat (Bio-Logic, Knoxville, TN, USA)
• EC-Lab® V11.02 software (Bio-Logic, Knoxville, TN, USA)
• RE-5B Ag/AgCl (3 M NaCl) reference electrode (BASi, Part No. MF-2052, West Lafayette, IN, USA)
• Ag/AgCl ink (Engineered Conductive Materials, Inc., Part No. CI-4001, Delaware, OH)
• Pt auxiliary electrode (BASi, Part No. MW-4130, West Lafayette, IN, USA)
• Hook test lead wires (E-Z Hook, Arcadia, CA, USA)
• L-glutamic acid, 99+% (Alfa Aesar, Stock # A15031, Tewksbury, MA, USA)
• L-ascorbic acid, 99% (Sigma-Aldrich, Product # A92902, St. Louis, MO, USA)
• Phosphate-buffered saline (PBS), 10X (0.1 M) pH 7.4 (Thermo Fisher Scientific, Cat. # AM9625, Waltham, MA, USA)
• Cimarec+™ stirring hotplate (Thermo Fisher Scientific, Cat. # SP88857107, Waltham, MA, USA)
• 1 Mil Kapton® tape (polyimide tape) (Kapton Tape, Part # KPT-1/4, Torrance, CA, USA)
• Krebs solution (124 mM NaCl, 2 mM KCl, 1.24 mM KH2PO_4_, 26 mM NaHCO_3_, 10 mM ascorbic acid, 1.3 mM MgSO_4_, 1.2 mM CaCl_2_, 10 mM glucose, bubbled continuously with 95% O2, 5% CO_2_, to maintain pH 7.2–7.4)
• Ketamine
• Xylazine
• Male Sprague-Dawley rat, 200–400 g (Envigo RMS, Inc., Indianapolis, IN)
• Double sucrose gap recording chamber (Fig. 2)
• Micromanipulator (Newport, 433 series, Irvine, CA, USA) (Fig. 2)
• Minipuls 3 peristaltic pump (Gilson, Middleton, WI, USA)
• Fine point forceps (Thermo Fisher Scientific, Cat. # 12-000-122, Waltham, MA, USA)

### Protocol

**Fig. 1:**
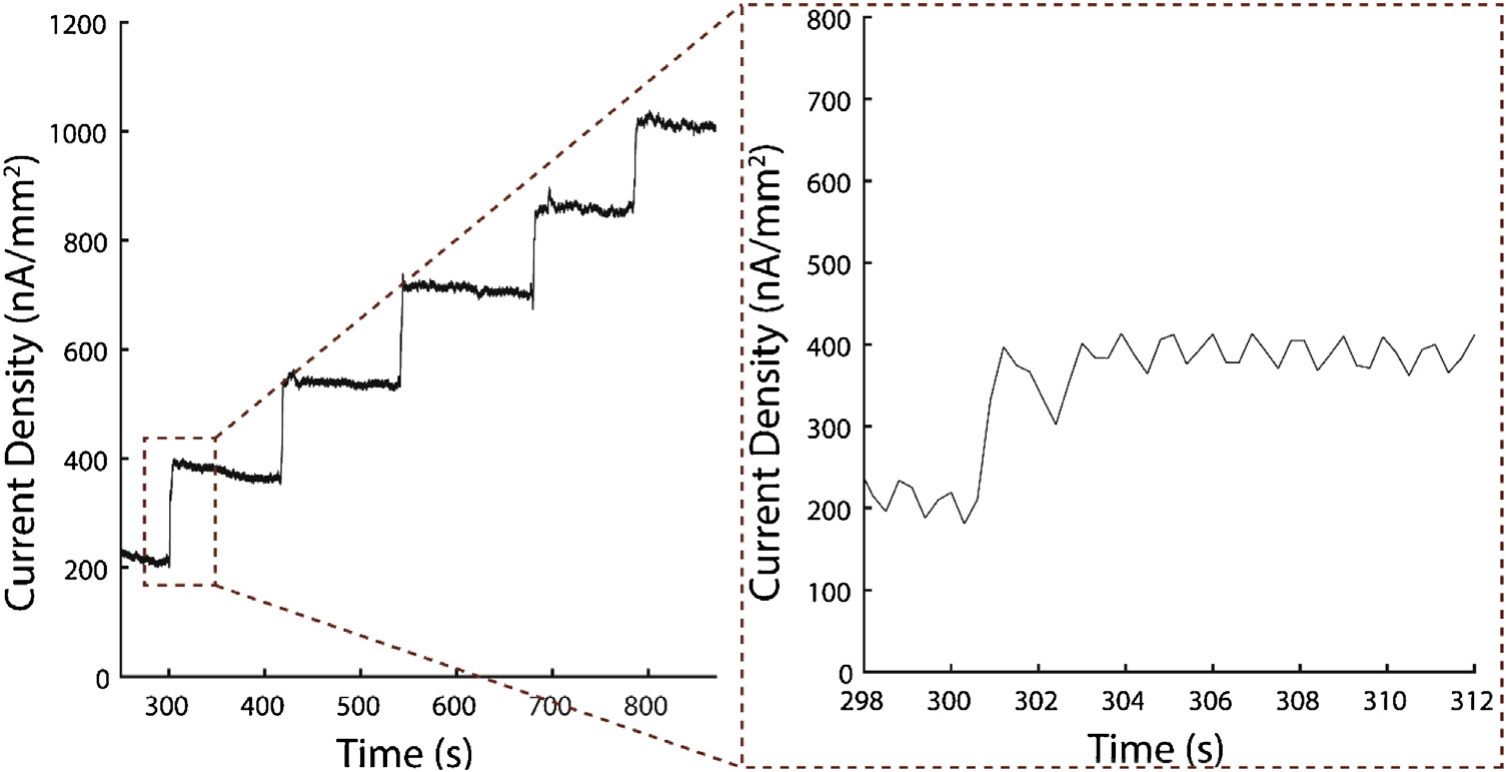
Current density vs. time during calibration of our glutamate biosensor prior to ex-vivo measurement (50, 100, 150, 350, and 550 μM glutamate). The insert to the right contains the signal step from addition of 50 μM glutamate, which shows the fast response of our glutamate biosensors.
  h When finished, wash the glutamate biosensor with fresh 0.01 M PBS and store the biosensor in 0.01 M PBS before use later in the protocol.
    Notes
2 Information on our glutamate biosensor is included in the supplementary material section.
3 If using a different glutamate biosensor with this protocol, make sure that it also is selective against ascorbic acid [1,5,6].
4 The staircase current response of the biosensor to glutamate is used to create a calibration line of sensitivity to glutamate.
5 Surgically extract spinal cord [2].
  a Anesthetize the animal with a mixture of ketamine (80 mg/kg) and xylazine (10 mg/kg) through intraperitoneal injection.
  b Transcardially perfuse the animal with cold oxygenated Krebs solution.
  c Rapidly remove the vertebrae column.
  d Perform complete laminectomy.
  e Carefully cut spinal roots to isolate the spinal cord.
  f Remove the dura mater to make insertion of the glutamate biosensor easier.
  g Subdivide the spinal cord twice longitudinally to obtain ventral white matter strips (spinal cord segments).
    *Note:* We used animals under strict accordance to the Purdue University Animal Care and Use Committee protocol and guidelines.
6 Incubate spinal cord segments in fresh Krebs solution for 60 min at room temperature.
  *Note:* Spinal cord segments can be kept Krebs solution perfused with O_2_ for up to 4 h.
7 Place spinal cord segment into a double sucrose gap recording chamber (Fig. 2, Fig. 3a).
  a Place the spinal cord segment across the central compartment, sucrose gap compartments and outside wells.
  b Continuously perfuse central compartment with 2 mL/min 37 °C oxygenated Krebs solution using peristaltic pump.

1 Calibrate biosensor [1] to glutamate.
  a Prepare 50 mL 0.01 M PBS in a 100-mL beaker and heat to 37 °C.
  b Add magnetic stir bar to PBS and stir at about 200 rpm.
  c Place biosensor, RE-5B Ag/AgCl reference electrode and auxiliary electrode in the PBS.
  d Apply 0.5 V to the biosensor (working electrode) versus the reference electrode (amperometry).
  e Wait at least 20 min after applying the 0.5 V potential for non-Faradaic current to decrease.
  f Add 50 μM glutamate (50 μL of 50 mM glutamate) three times and wait for the current to settle between each time.
  g Add 200 μM glutamate (200 μL of 50 mM glutamate) two times and wait for the current to settle between each time. The result of steps 1f–g is a five-point calibration at 50, 100, 150, 350 and 550 μM glutamate) (Fig. 1).

**Fig. 2:**
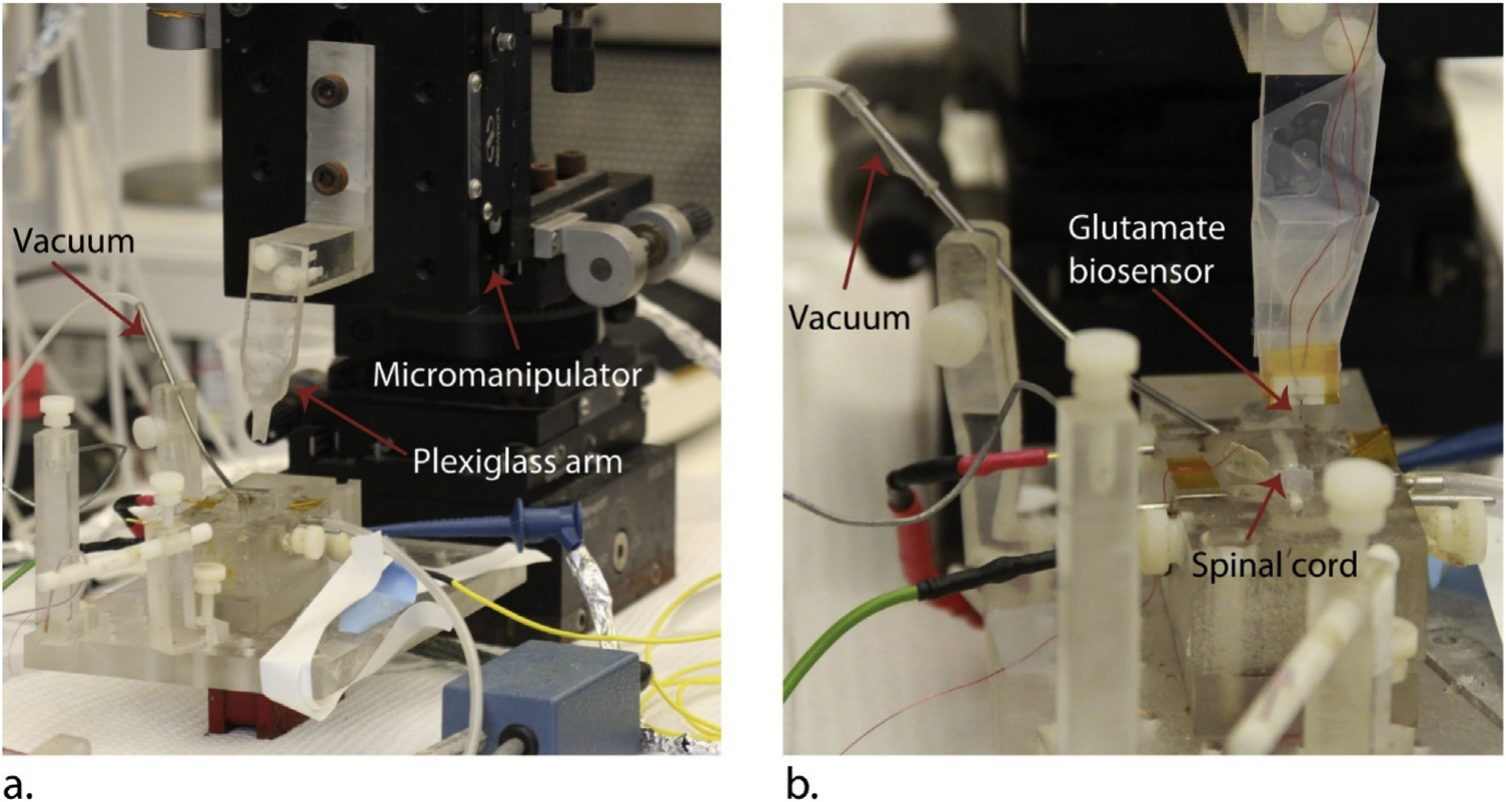
(a) Photograph of double sucrose gap recording chamber, Plexiglass arm for holding the glutamate biosensor and the micromanipulator for controlling vertical movement of the glutamate biosensor. The glutamate biosensor is not attached to the arm in this photograph. (b) The glutamate biosensor has been attached to the plexiglass arm, and a white matter strip (spinal cord) has been placed in the sucrose gap recording chamber.

**Fig. 3:**
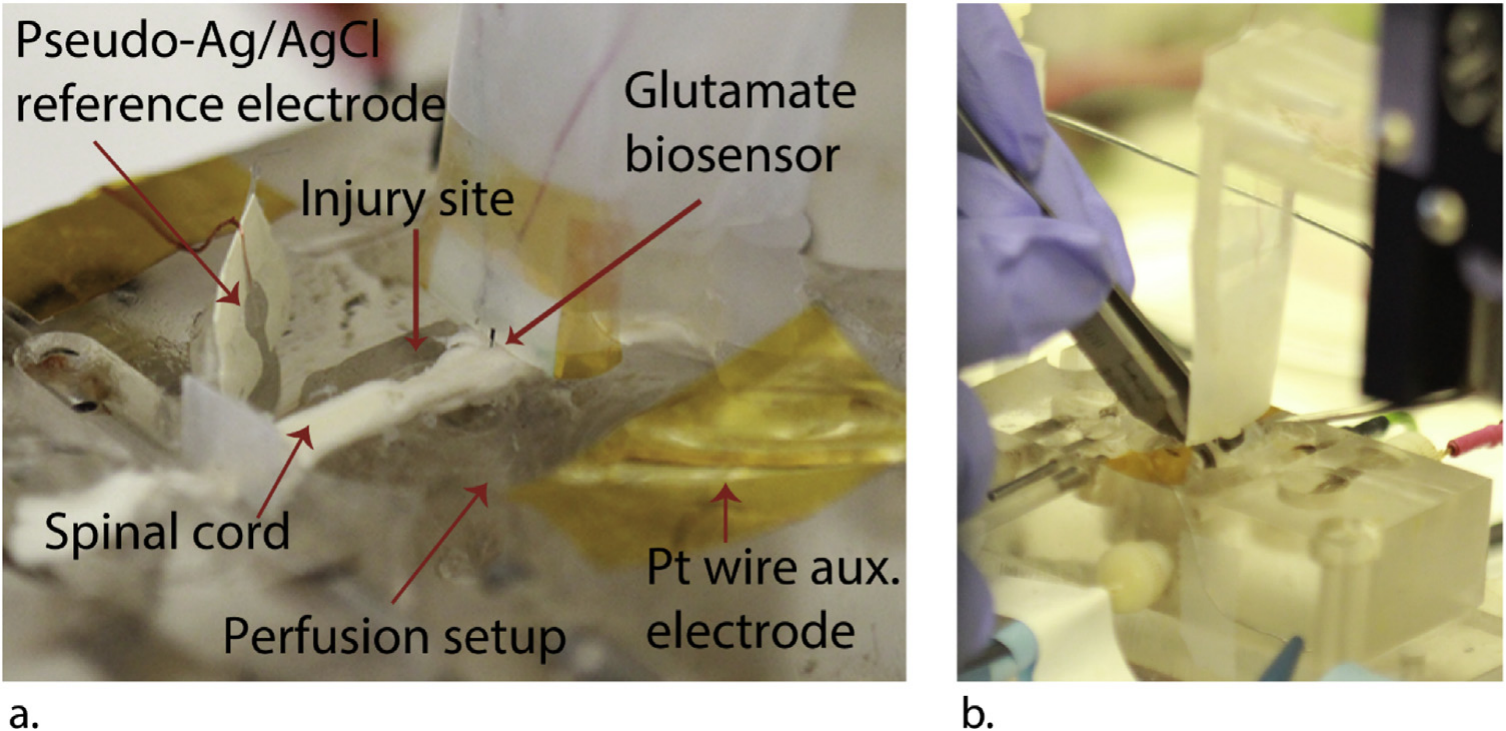
(a) Photograph of glutamate biosensor inserted into a rat spinal cord segment in a double sucrose gap recording chamber. Important components of the system are labeled. (b) Photograph of compressing the spinal cord with forceps to simulate compression injury.
8 Mount a pseudo-Ag/AgCl reference electrode [1] and Pt auxiliary electrode to the sidewalls of the central compartment of the recording chamber, so they sick into the Krebs solution as shown in Fig. 3a.
  Notes:
9 Information on making a pseudo-Ag/AgCl electrode is included in the supplementary material section.
10 We measured −76 mV as the potential of the pseudo Ag/AgCl electrode vs. the BASi RE-5B Ag/AgCl reference electrode.
11 Polyimide tape was used to secure the pseudo-Ag/AgCl reference and auxiliary electrodes to the recording chamber.
12 Attach glutamate biosensor (working electrode) to the Plexiglass arm above the recording chamber with tape.
13 Using a micromanipulator, lower the Plexiglass arm with the glutamate biosensor attached (Fig. 2), so the glutamate biosensor sticks 1–1.5 mm into the spinal cord segment.
  *Note:* Although the 50-μm thick liquid crystal polymer biosensor shank is flexible compared to silicon and ceramic, we were able to insert these shanks 1–1.5 mm into the spinal cord segment. We tested implantation before ex-vivo implantation with 0.6% agarose gel, a model for device insertion into brain tissue [7].
14 Connect working, reference and counter electrodes to the potentiostat with test hook clips.
15 Apply +0.5 V to the working electrode versus the pseudo-Ag/AgCl reference electrode and record current.
  *Note:* The choice of holding potential depends on the electrochemical sensor used. Applying +0.5 V vs. Ag/AgCl is an adequate holding potential oxidase/Pt-based electrochemical biosensors [1,8,9]. Another holding potential typically used for this class of biosensors is +0.7 V vs. Ag/AgCl.
16 Wait at least 20 min (1200 s) after applying the 0.5 V potential for non-Faradaic current to decrease.
17 At 20 min (1200 s), simulate spinal cord injury by compressing about 70 N with forceps for 5 s at the part of the spinal cord segment immediately in front of where the glutamate biosensor is inserted (Fig. 3b).
  Notes:
18 70 N corresponds to about 70% of one’s maximum pinching force [10].
19 Compressing the spinal cord for more than 10 s risks breaking it in two.
20 For comparison, at 40 min (2400s) use a micropipette to inject 100 μL 50 mM glutamate at the site of injury.

### Protocol validation

**Fig. 4:**
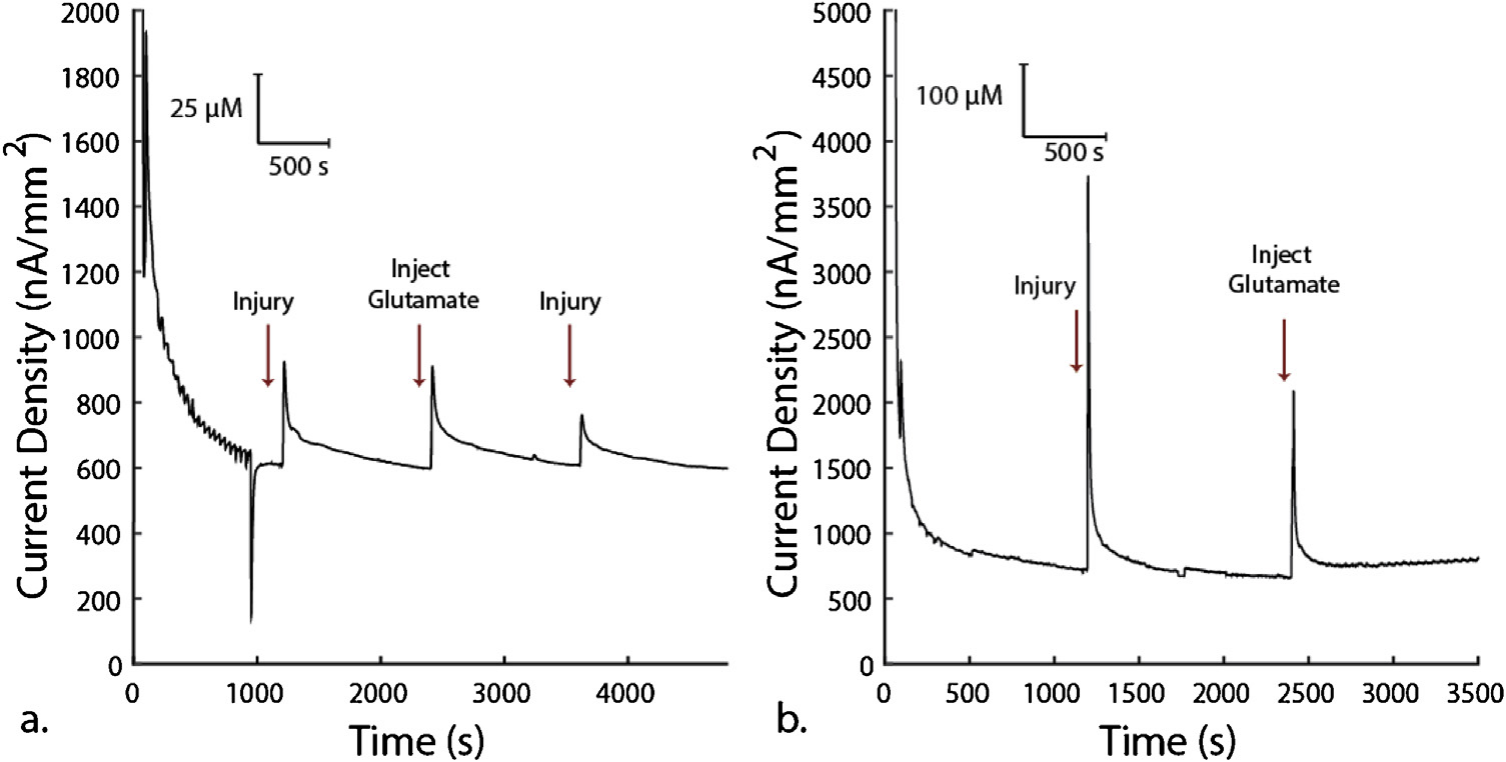
Validation of *ex vivo* glutamate measurement following injury in spinal cord segment of a rat. The printed micro-scale glutamate biosensors were inserted into spinal cord white matter strips before injury. At 20 min (1200 s) we injured the spinal cord by compressing it about 70 N (70% max strength) with forceps for 5 s at the part of the spinal cord segment immediately in front of where the glutamate biosensor is inserted. At 40 min (2400 s) we used a micropipette to inject 100 μL or 50 mM glutamate at the same location as injury. **(a)** At 60 min (3600 s) we repeated injury at the same location. Each subfigure shows an independent experiment from a different white matter strip from the same animal on the same day.

Using this method, we measured spikes in glutamate concentration following injury of half segment of rat spinal cord *ex vivo*. Fig. 4 shows these measurements.
