# Supplementary material for: Ex vivo electrochemical measurement of glutamate release during spinal cord injury
Source: MethodsX. 2019 Aug 23;6:1894–900. doi: 10.1016/j.mex.2019.08.008 (PMC6727010; doi:10.1016/j.mex.2019.08.008)
Supplement: Supplementary file 1 [file mmc1.docx]

**Supplementary material:**

*Glutamate Biosensor*

The glutamate biosensor was made on a liquid crystal polymer shank (250 µm wide, 50 µm thick) with a nanocomposite “working” electrode printed on top (100 µm) (Fig. S1). The nanocomposite was a mix of PEDOT:PSS, Ecoflex™ silicone rubber, multi-walled carbon nanotubes and platinum nanoparticles. The nanocomposite working electrode was functionalized with glutamate oxidase enzyme cross-linked with glutaraldehyde and bovine serum albumin. A reference electrode and counter electrode were needed to bias the sensor and measure currents corresponding to glutamate concentration. The reference electrode was made of nanocomposite on liquid crystal polymer functionalized with Ag/AgCl ink. The counter electrode was a Pt wire. Another work of ours elaborates the fabrication, characterization (including selectivity) and operation of this glutamate biosensor [1].


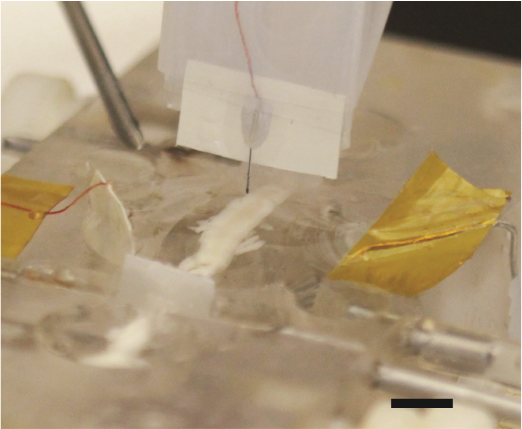


Figure S1. Photograph of the glutamate biosensor before insertion into spinal cord segment, scale bar = 5 mm. The glutamate biosensor was printed onto a 250-µm wide shank.

*Pseudo-Ag/AgCl Reference Electrode*

We made pseudo-Ag/AgCl reference electrodes by printing Ag/AgCl ink on top of nanocomposite traces on liquid crystal polymer. Then, we applied magnet wire (stripped of insulating coating on ends) to the other end of the nanocomposite trace with Ag ink. We then insulated the conductive trace with PDMS, so only the Ag/AgCl electrode is exposed [1]. Alternatively, a pseudo-Ag/AgCl reference electrode can be made by electroplating Cl onto a Ag wire with 9-15 VDC in 50 g NaCl/90 ml 1 M HCl. The plating reaction is Ag + Cl^-^ 🡪 AgCl + e^-^.

*Data Analysis*

Amperometry data recorded from the glutamate biosensor with EC-Lab® V11.02 software was plotted using MATLAB, and figures were formatted with Adobe Illustrator. Other software may be used depending on the potentiostat.

*Potential Improvement to the Method*

A sentinel electrode / control electrode could be used in combination with the glutamate biosensor with a multiplexer or multi-channel potentiostat [2,3]. The sentinel electrode would measure background and noise currents that could be subtracted from glutamate biosensor signal. A sentinel electrode would be made on the same shank as the glutamate biosensor and functionalized with only BSA and glutaraldehyde and not with glutamate oxidase. Further functionalizing the glutamate biosensors with ascorbate oxidase, which catalyzes ascorbate into dehydroascorbate and water, would further eliminate interference from ascorbate and improve biosensor selectivity [4]. Finally, calibrating each sensor to ascorbate before ex-vivo measurement would ensure that all measurements are selective for glutamate.

We expect differences in the depth of implantation, the compression by forceps, and the relative distance between sensor and injury/injection sites to have caused variability in glutamate signal during ex-vivo measurement (Fig. 4). This variability may be minimized by using micromanipulators and SCI inducing tools (e.g., the NYU impactor [5]) to better control the location and the amount of the injury or injection.

**References:**

[1] T.N.H. Nguyen, J.K. Nolan, H. Park, S. Lam, M. Fattah, J.C. Page, H.-E. Joe, M.B.G. Jun, H. Lee, S.J. Kim, R. Shi, H. Lee, Facile fabrication of flexible glutamate biosensor using direct writing of platinum nanoparticle-based nanocomposite ink, Biosens. Bioelectron. 131 (2019) 257–266. https://doi.org/10.1016/J.BIOS.2019.01.051.

[2] J.J. Burmeister, G.A. Gerhardt, Self-referencing ceramic-based multisite microelectrodes for the detection and elimination of interferences from the measurement of l-glutamate and other analytes, (2001). https://doi.org/10.1021/AC0010429.

[3] N.R. Ferreira, A. Ledo, J. Laranjinha, G.A. Gerhardt, R.M. Barbosa, Simultaneous measurements of ascorbate and glutamate in vivo in the rat brain using carbon fiber nanocomposite sensors and microbiosensor arrays, Bioelectrochemistry. 121 (2018) 142–150. https://doi.org/10.1016/J.BIOELECHEM.2018.01.009.

[4] M. Ganesana, E. Trikantzopoulos, Y. Maniar, S.T. Lee, B.J. Venton, Development of a novel micro biosensor for in vivo monitoring of glutamate release in the brain, Biosens. Bioelectron. 130 (2019) 103–109. https://doi.org/10.1016/J.BIOS.2019.01.049.

[5] J.A. Gruner, A monitored contusion model of spinal cord injury in the rat, J. Neurotrauma. 9 (1992) 123–128. https://doi.org/10.1089/neu.1992.9.123.
